# T Cell-Specific Overexpression of TGFß1 Fails to Influence Atherosclerosis in ApoE-Deficient Mice

**DOI:** 10.1371/journal.pone.0081444

**Published:** 2013-12-05

**Authors:** Kurt Reifenberg, Fei Cheng, Laura Twardowski, Ines Küpper, Elena Wiese, Franziska Bollmann, Hartmut Kleinert, Manfred Blessing, Karl J. Lackner, Michael Torzewski

**Affiliations:** 1 Animal Laboratory Services, German Cancer Research Center, Heidelberg, Germany; 2 Institute of Clinical Chemistry and Laboratory Medicine, University Medical Center, Johannes Gutenberg-University, Mainz, Germany; 3 Department of Laboratory Medicine, Robert-Bosch-Hospital, Stuttgart, Germany; 4 Central Laboratory Animal Facility, Johannes Gutenberg-University, Mainz, Germany; 5 Department of Pharmacology, University Medical Center, Johannes Gutenberg-University, Mainz, Germany; 6 Center for Biotechnology and Biomedicine, Veterinary Faculty, University of Leipzig, Leipzig, Germany; Klinikum rechts der Isar, Technische Universität München, Germany

## Abstract

Clinical data have indicated a negative correlation between plasma TGFß1 concentrations and the extent of atherosclerosis and have thus led to the hypothesis that the pleiotropic cytokine may have anti-atherogenic properties. T-cells are currently discussed to significantly participate in atherogenesis, but the precise role of adaptive immunity in atherogenesis remains to be elucidated. TGFß1 is known to strongly modulate the function of T-cells, however, inhibition of TGFß1 signalling in T-cells of atherosclerosis-prone knock-out mice failed to unequivocally clarify the role of the cytokine for the development of atherosclerosis. In the present study, we thus tried to specify the role of TGFß1 in atherogenesis by using the murine CD2-TGFß1 transgenic strain which represents a well characterized model of T-cell specific TGFß1 overexpression. The CD2-TGFß1 transgenic mice were crossed to ApoE knock-out mice and quantity and quality of atherosclerosis regarding number of macrophages, smooth muscle cells, CD3 positive T-cells and collagen was analyzed in CD2-TGFß1 ApoE double mutants as well as non-transgenic ApoE controls on both normal and atherogenic diet of a duration of 8, 16 or 24 weeks, respectively. In all experimental groups investigated, we failed to detect any influence of TGFß1 overexpression on disease. Total number of CD3-positive T-lymphocytes was not significantly different in atherosclerotic lesions of CD2-TGFß1 ApoE^−/−^ females and isogenic ApoE^−/−^ controls, even after 24 weeks on the atherogenic diet. The synopsis of these data and our previous study on TGFß1 overexpressing macrophages suggests that potential effects of TGFß1 on atherosclerosis are most probably mediated by macrophages rather than T-cells.

## Introduction

The pleiotropic cytokine TGFß1 can exert multiple immunomodulatory effects [1], [2] which is impressively reflected by the respective phenotype of knock-out mice, dying pre- or perinatally because of widespread inflammatory reactions [3], [4]. TGFß1 is synthesized by several cardiovascular cell types involved into atherogenesis including endothelial cells, monocytes/macrophages and T-cells [5]. The potential of TGFß1 to inhibit the proliferation of smooth muscle cells (SMCs) *in vitro* and to induce the formation of extracellular matrix [6], [7] has led to the assumption that this cytokine may have anti-atherogenic properties [8]. This hypothesis is supported by clinical studies indicating a negative correlation between plasma TGFß1 concentrations and the extent of atherosclerosis [8], [9], [10]. Experimental inhibition of TGFß1 in blood vessels of atherosclerosis-prone ApoE knock-out mice by specific antibodies [11] or recombinant soluble type II receptor [12] resulted in accelerated atherosclerosis or a more pro-inflammatory plaque phenotype and thus principally confirmed the putative anti-atherogenic TGFß1 properties. Further, transgenic TGFß1 overexpression in the heart and plasma of atherosclerotic mice led to reduction of atherosclerosis and thus also supported the atheroprotective function of TGFß1 [13]. In an attempt to more specifically address the mechanisms underlying the effects of TGFß1 on atherogenesis, we have recently shown that overexpression of TGFß1 in macrophages reduces and stabilizes atherosclerotic plaques in ApoE^−/−^ mice [14]. Concerning T cells, numerous studies have provided evidence for their involvement in atherosclerosis, although the number of this cell type in the lesion is sparse [15]. Goyova et al. [16] and Robertson and co-workers [17] used crossing experiments or bone marrow transplantation to introduce transgenic T-cells into atherosclerosis-prone mice in which the TGF-ß1 signal transduction was specifically inhibited by overexpression of a dominant negative form of the type II receptor. Although these two studies found opposite effects on lesion size, they were in general agreement that blockade of TGF-ß signalling in T cells increased vascular inflammation (which itself is certainly atherogenic). However, the experimental approach of T-cell specific inhibition of TGF-ß1 signalling by overexpression of a dominant negative type II receptor must generally be considered as limited since the immunological phenotype of such mouse strains differs depending on expression pattern and promoters used [16], [17] and is significantly more moderate compared to mice where the TGFBR2 gene was T-cell specifically targeted using the conditional Cre/LoxP technology [18].

Stimulated by the experimental results of T-cell specific inhibition of TGFß1 signalling during atherogenesis [16], [19], the aim of the present study was to reciprocally investigate the influence of T-cell specific TGFß1 overexpression on atherosclerotic disease. To this end we used the CD2-TGFß1 transgenic mouse strain which represents a well characterized murine model of TGFß1 overexpression and activated regulatory T-cells [20], [21] previously shown to suppress disease development in murine models of colon cancer [22] and airway hyper-reactivity [23]. In comparison to non-transgenic controls, isolated CD3-positive splenocytes of CD2-TGFß1 transgenic mice express active TGFß1 and synthesize higher levels of TGFß1 upon stimulation with an anti-CD3/CD28 mAb. By using an autocrine mechanism T-cell specific transgenic TGFβ1 overexpression leads to an increased frequency of CD4+CD25+ cells and an overexpression of the Treg-specific transcription factor Foxp3 in the thymus [21]. We crossed these transgenics to the atherosclerotic ApoE knock-out strain and quantitatively analyzed the atherosclerotic lesions of the resulting double mutants.

## Materials and Methods

### Transgenic Mice and Dietary Amplification of Atherosclerosis

Transgenic strain CD2-TGFß1-Y ([21], internal strain designation CD2-TGFß1, official strain designation TgN (CD2-TGFß)3Mbl) carries the cDNA of a mutated simian TGFß1 gene [24] under the transcriptional regulation of the human CD2 promoter element [25]. The mutation consists of the insertion of two serine residues at positions 223 and 225 of the simian cDNA leading to the expression of a biologically active TGFß1 form. CD2-TGFß1 transgenic mice have been shown to efficiently overexpress the TGFß1 cytokine in T-cells [20], [21]. The strain was provided on a C57BL/6 inbred background. CD2-TGFß1 transgenic mice could be discriminated from their non-transgenic littermates by PCR amplification of specific sequences using primers 5′-TTT GTA GCC AGC TTC CTT CTG-3′ and 5′-TCG ATA GTC TTG CAG GTG GAT-3′.

For generation of atherosclerotic CD2-TGFß1 transgenic mice hemizygous transgenics were crossed to B6.129P2-Apoe^tm1Unc^/J (ApoE) knock-out mice (The Jackson Laboratory, Bar Harbour, Maine, USA; Stock No 2052). Transgenic atherosclerotic experimental animals as well as non-transgenic controls were generated by backcrossing the CD2-TGFß1 ApoE^−/−^ double-mutant mice to ApoE^−/−^ animals. Murine ApoE genotypes were determined by PCR as recommended in the protocol provided by The Jackson Laboratory.

Female transgenic CD2-TGFß1 ApoE^−/−^ experimental mice as well as non-transgenic ApoE^−/−^ controls were raised and fed under identical conditions until an age of 8 weeks. Thereafter, the animals were either administered a normal mouse chow diet (ND, Ssniff Spezialdiäten GmbH, Soest, Germany) or an atherogenic Western type diet (WTD, Ssniff Spezialdiäten GmbH, Soest, Germany) for 8, 16 or 24 weeks, respectively. The WTD contained 21% (wt/wt) fat and 0.15% (wt/wt) cholesterol.

All laboratory mice were maintained at the Central Laboratory Animal Facility of the University of Mainz under strict SPF (specific pathogen free) conditions. Animals were housed in accordance with standard animal care requirements and maintained on a 12/12 hour light-dark cycle. Water and food were given *ad libitum*. Genetic authenticity of all strains was monitored commercially (KBioscience, Hoddesdon, UK) using the SNP-based marker set previously developed by The Jackson Laboratory [26].

All animal work performed in this study was conducted according to the national guidelines and was reviewed and confirmed by an institutional review board/ethics committee headed by the local animal welfare officer (Prof. Kempski) of the University Medical Center (Mainz, Germany). The animal experiments were finally approved by the responsible national authority, which is the National Investigation Office Rheinland-Pfalz (Koblenz, Germany). The Approval ID assigned by this authority is AZ 23 177-07/G 07-1-003.

### Lipoprotein Analysis of Murine Sera

Murine sera were diluted 1∶3 before quantitative cholesterol and triglyceride analyses. Quantitative cholesterol determinations were conducted using a colorimetric assay (CHOD-PAP, Roche Diagnostics, Mannheim, Germany). Triglycerides were determined by quantifying free glycerine originating from hydrolytic cleavage (GPO-PAP, Roche Diagnostics).

### Tissue Preparation and Quantification of Atherosclerotic Lesions

Hearts and aortas of sacrificed mice were resected and fixed with formaldehyde *en bloc*. Longitudinal sections of the aortic arch were stained with trichrome and plaque size quantitated as described previously [27], [28]. The rest of the aortae from the arch down to the iliac bifurcation was opened longitudinally and stained with freshly prepared Sudan IV. Lesions *en face* were quantified using Photoshop-based image analysis as described [28].

### Immunohistochemical and Histochemical Analyses

Serial 5 µm-thick sections of the paraffin-embedded aortic arch were deparaffinised in xylene. All slides were treated with 3% H_2_O_2_ to block endogenous peroxidase activity. Slides were incubated consecutively with 5% normal serum to block non-specific binding, primary antibodies rat anti-mouse F4/80 antibody (clone CI:A3-1, 1∶100, Acris Antibodies), murine anti-smooth muscle α-actin (1A4, 1∶100, Sigma) and rat anti-CD3 (CD3-12, 1∶1000, Acris Antibodies), respectively, for 1 hour, biotin-conjugated secondary anti-mouse or anti-rat antibody for 30 minutes and avidin-biotin-peroxidase reagent for 45 minutes at room temperature. Immunostaining of murine tissues with the murine MAb was performed using the Vector M.O.M. immunodetection kit (Vector Laboratories). Prior to staining with rat anti-CD3 antibody, antigen retrieval using heat treatment for 20 min (1 mM EDTA, pH 8.0) was performed. The reaction products were identified by immersing the slides in diaminobenzidine tetrachloride (DAB) to give a brown reaction product. The slides were then counterstained with hemalaun and mounted. Negative controls included replacement of the primary antibody by irrelevant isotype-matched antibodies. Collagen content was analysed by picrosirius red and polarized light microscopic imaging. Percent-positive area for immunohistochemical or picrosirius red staining was quantified by Photoshop-based image analysis as described [28], [29], [30]. Briefly, pixels with similar chromogen characteristics were selected with the “magic wand” tool and the “select similar” command, and the ratio of the positively stained area to the total lesion area studied was calculated with the “histogram” command in Photoshop. All quantitative morphometric and immunohistochemical data were collected independently by two experienced operators blinded to the mice genotypes.

### Statistical Analyses

Data were analysed with SPSS 17.0 for Windows. Most of the outcome parameters determined in this study (atherosclerosis *en face*, macrophage, T-cell and smooth muscle cellularity as well as collagen content of atherosclerotic lesions) did not follow a normal distribution as judged by Shapiro-Wilk tests. These parameters are thus presented as box-plots with median, interquartile range, minimum and maximum diagrams and their statistical analyses have been performed with the non-parametric Mann-Whitney *U* tests. Body weights and serum lipid concentrations have been found to follow a normal distribution. These data are thus presented as mean (± standard deviation) and have been analyzed by t-test of significance. Differences between the mice genotypes were considered as significant for p-values <0.05.

## Results

### Serum Lipids and Lipoproteins

Female CD2-TGFß1 ApoE^−/−^ experimental mice and isogenic ApoE^−/−^ littermate controls were generated according to the breeding schedule mentioned in the Materials and Methods' section and starting at an age of 8 weeks all mice were either administered a ND or an atherogenic WTD for another 8, 16 or 24 weeks, respectively. At the end of the diet all animals were sacrificed for biochemical serum analyses as well as quantitative and qualitative characterization of atherosclerotic lesions. Table 1 shows the group sizes, body weights, serum cholesterol levels and serum triglyceride concentrations of CD2-TGFß1 ApoE^−/−^ mice and ApoE^−/−^ controls of the various dietary groups. Except for body weights after 24 weeks on ND and serum cholesterol levels after 8 weeks on ND, no significant differences of these parameters could be detected in the experimental groups, indicating the balance between transgenic and non-transgenic ApoE^−/−^ mice with regard to body weight and serum lipids. Due to the significant differences regarding serum cholesterol levels but in particular with regard to the sparse atherosclerotic lesion development of these groups, mice administered a ND for 8 weeks were excluded from further analyses.

**Table 1:** Lipoprotein analysis of murine sera.

	group size		body weight, g		serum cholesterol, mg/dL		serum triglycerides, mg/dL	
diet	CD2-TGFß ApoE^−/−^	ApoE^−/−^	CD2-TGFß ApoE^−/−^	ApoE^−/−^	CD2-TGFß ApoE^−/−^	ApoE^−/−^	CD2-TGFß ApoE^−/−^	ApoE^−/−^
8 w ND	9	10	22,4±5,5	23,4±3,5	256 **±26	319±51	156±46	175±76
16 w ND	10	10	25,6±2,5	25,8±1,8	326±40	333±21	138±24	157±37
24 w ND	10	10	25,2 *±2,4	29,1±3,6	291±29	329±111	164±161	216±182
8 w WTD	10	10	27,8±2,4	25,7±2,8	1562±159	1722±235	28,5±10,2	26,2±15,7
16 w WTD	10	10	29±3,9	30,2±3,9	1378±524	1515±270	28,1±11,9	33,4±15,7
24 w WTD	11	13	28,4±4,4	31,7±7,1	1143±356	1378±270	23,4±9,6	22,5±10,1

Group size, body weights, serum cholesterol and serum triglyceride concentrations of CD2-TGFß1 ApoE^−/−^ and ApoE^−/−^ mice on ND and WTD. Data are presented as means ± standard deviations.

*, ** indicate statistically significant differences (* p<0,05, ** p<0,01).

### Atherosclerosis Lesion Progression

**Figure 1:**
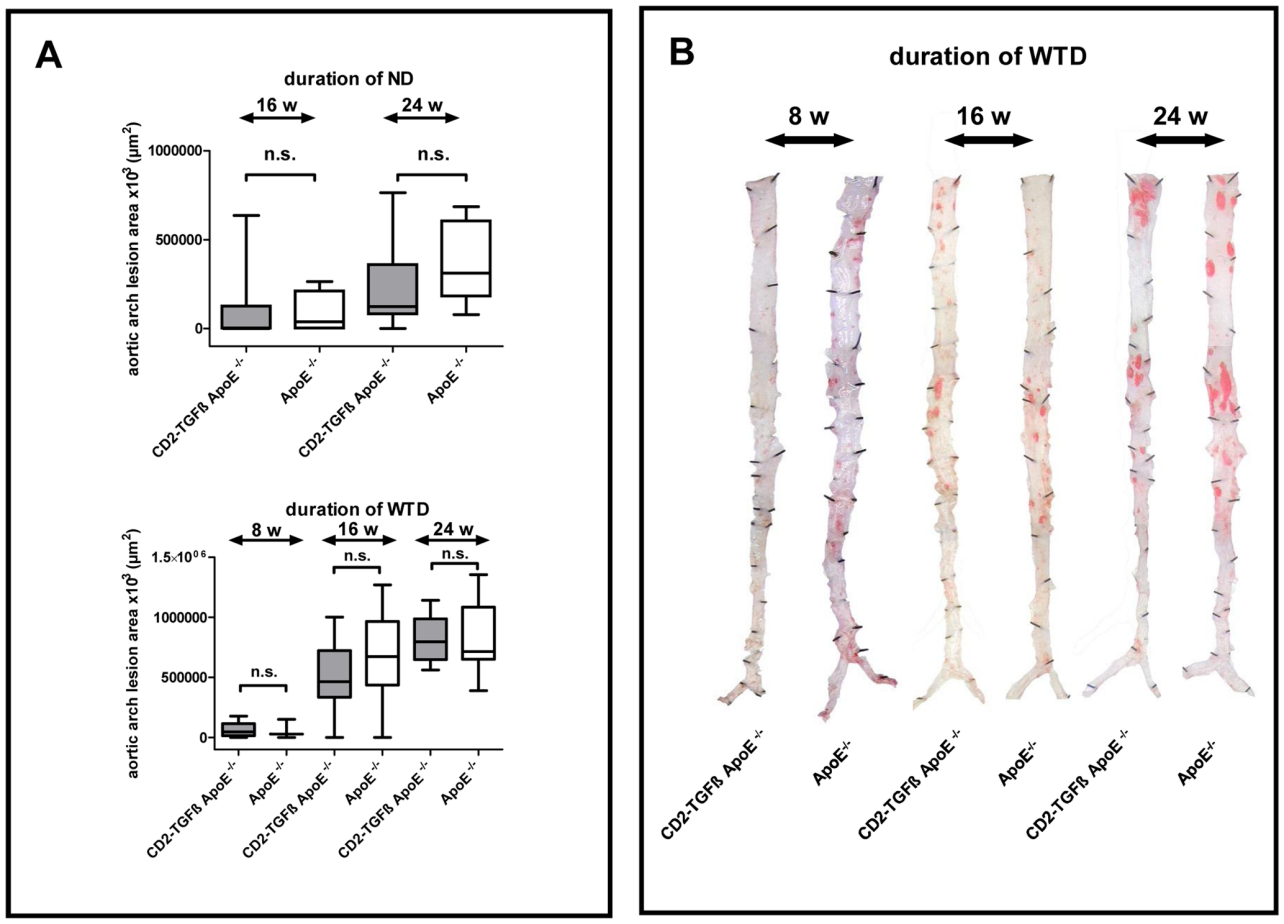
Quantification of atherosclerosis in the mouse model of TGFß1 overexpressing T cells. **A**, Box and whisker diagrams (median, interquartile range, minimum, and maximum; n.s.  =  not significant) of the maximal area of the inner aortic arch intima (lesser curvature) of CD2-TGFß1 ApoE^−/−^ and ApoE^−/−^ mice on ND (upper panel) and WTD (lower panel). **B**, Representative Sudan-stained aortas *en face* of CD2-TGFß1 ApoE^−/−^ mice and ApoE^−/−^ controls on WTD.

In our next experiment we compared the atherosclerotic lesion size of TGFß1 overexpressing ApoE^−/−^ animals and non-transgenic controls. As shown in Fig. 1A, quantification of the maximal area of the inner aortic arch intima (lesser curvature) failed to reveal significant differences in control mice compared to double-mutant mice, neither after 16 and 24 weeks on ND nor after 8, 16 and 24 weeks on WTD. Likewise, we failed to detect any significant differences of atherosclerosis *en face* in all dietary groups analyzed (data not shown). The lesional distribution pattern of CD2-TGFß1 ApoE^−/−^ mice and ApoE^−/−^ controls was comparable for all dietary periods investigated (Fig. 1B).

### Phenotype Analysis of Atherosclerotic Lesions

**Figure 2:**
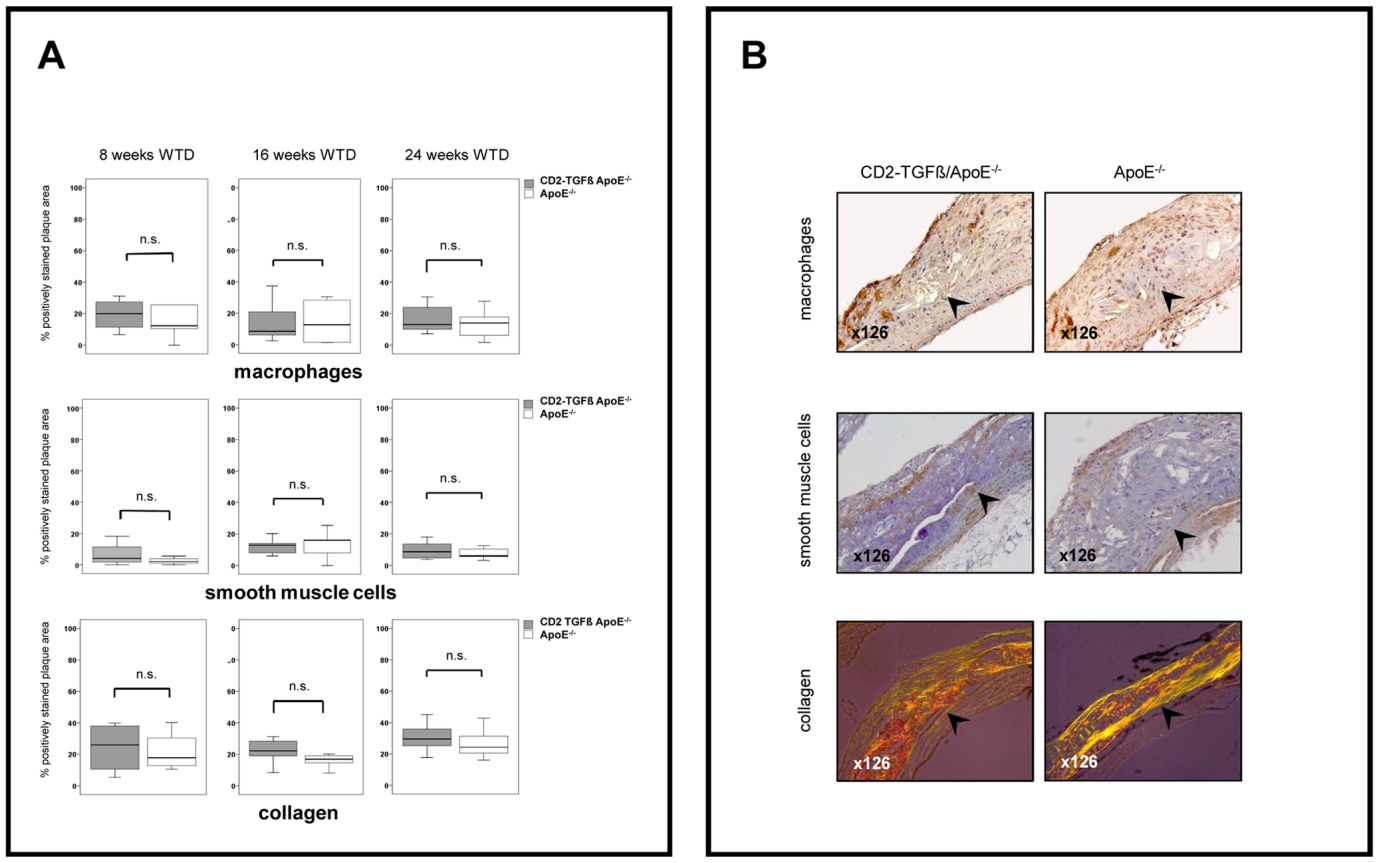
Lesion composition of atherosclerotic CD2-TGFß1 ApoE^−/−^ and ApoE^−/−^ mice. **A**, Box and whiskers diagrams (median, interquartile range, minimum, and maximum; n.s.  =  not significant) of the quantification of macrophages (upper panel), SMCs (middle panel) and collagen (lower panel) in atherosclerotic lesions of CD2-TGFß1 ApoE^−/−^ females and isogenic ApoE^−/−^ controls after 8, 16 and 24 weeks on WTD, respectively. **B**, Representative histological slides of atherosclerotic lesions located in the inner aortic arch intima (lesser curvature) of CD2-TGFß1 ApoE^−/−^ mutants and ApoE^−/−^ controls after 24 weeks on WTD. The slides have been stained for macrophages, SMCs, and collagen by using a rat anti-mouse F4/80 antibody (upper panel), a mouse anti-smooth muscle α-actin antibody (middle panel), and picrosirius red with subsequent polarization (lower panel), respectively. Percent-positive area for macrophages (upper panels, brown stained areas), SMCs (middle panels, brown-stained areas), and collagen (lower panels, areas with yellow, green, orange, or red polarized colour) were quantified by Photoshop-based image analysis. The aortic lumen is to the upper left corner. The demarcation between intima and media is indicated by arrowheads.

**Figure 3:**
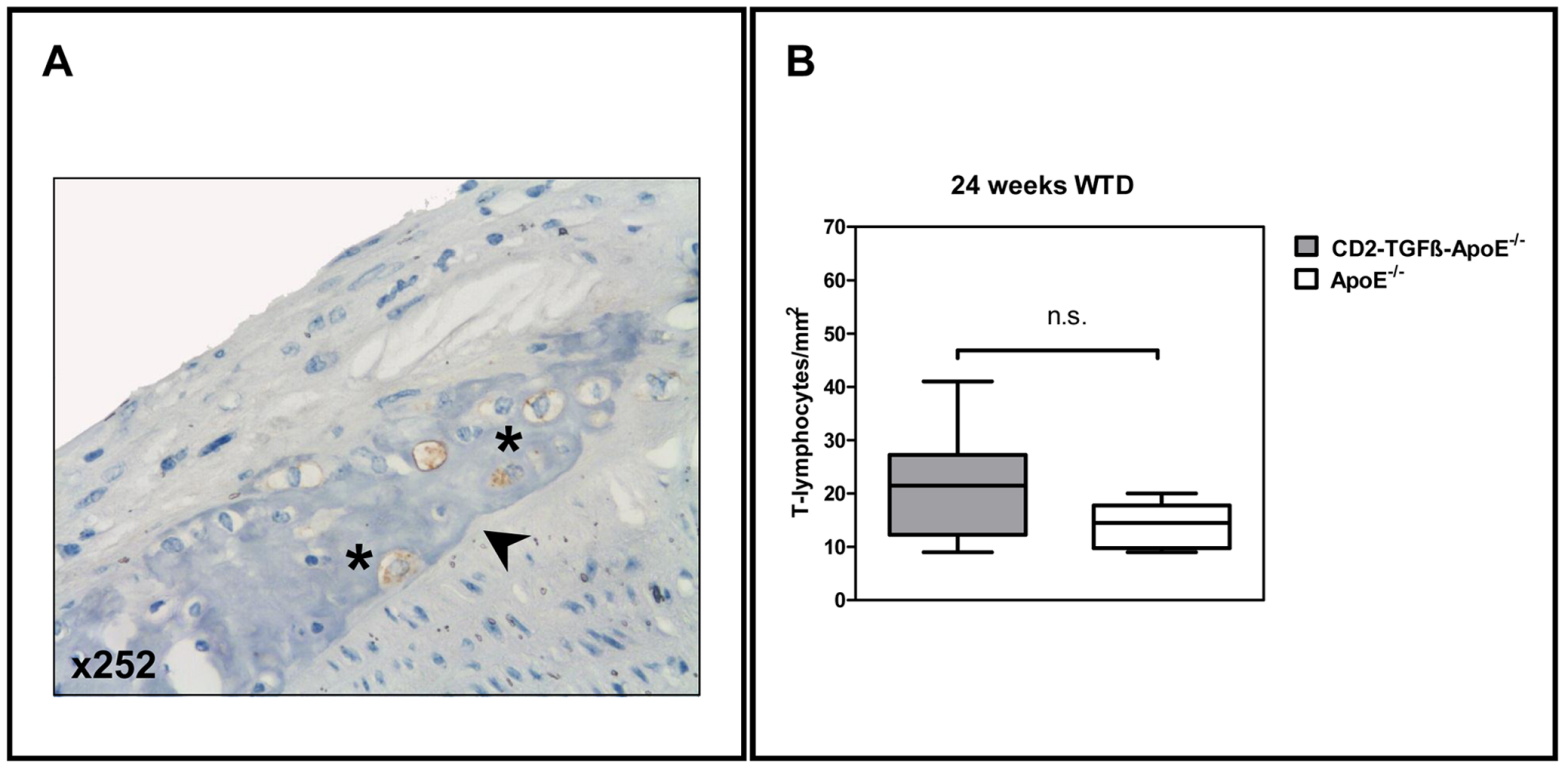
T-lymphocytes in atherosclerotic lesions. **A**, Representative immunohistochemical staining of an atherosclerotic lesion located in the inner aortic arch intima (lesser curvature) of a CD2-TGFß1 ApoE^−/−^ mouse after 24 weeks on WTD. The slide was stained for T-lymphocytes with a monoclonal antibody against CD3 (clone CD3-12, AbD Serotec MorphoSys AbD GmbH, Düsseldorf, Germany) and the number of positively stained cells (asterisks) per mm^2^ was counted (see **B**). The aortic lumen is to the upper left corner. The demarcation between intima and media is indicated by an arrowhead. **B**, Box and whiskers diagrams (median, interquartile range, minimum, and maximum) of the quantification of T-lymphocytes in atherosclerotic lesions of CD2-TGFß1 ApoE^−/−^ females and isogenic ApoE^−/−^ controls after 24 weeks on WTD (n.s.  =  not significant).

Since T-cell specific TGFß1 overexpression had no influence on atherosclerosis lesion progression, our next analyses were aimed to detect potential qualitative effects of transgenic TGFß1 overexpression on atherogenesis. To this end we compared lesion composition (mean percent of macrophages, smooth muscle cells (SMCs), T-cells, and collagen per aortic area) between the CD2-TGFß1 transgenic and non-transgenic ApoE^−/−^ mice (Figs. 2 and 3). For all dietary groups investigated we failed to detect any significant influence of T-cell specific TGFß1 overexpression on macrophage and SMC cellularity as well as on collagen contents of ApoE^−/−^ atherosclerosis (Fig. 2). Lesional cellularity of CD3+ T-cells was exemplarily investigated in ApoE^−/−^ mice on WTD after 24 weeks. However, no significant differences could be detected between CD2-TGFß1 transgenic mice and non-transgenic controls (Fig. 3).

## Discussion

Strong clinical evidence [31], [32] and animal experimental confirmation [14], [33], [34], [35] have been provided for an anti-atherogenic effect of the pleiotropic cytokine TGF-ß on atherogenesis. We have recently shown that overexpression of TGFß1 in macrophages reduces and stabilizes atherosclerotic plaques in ApoE^−/−^ mice [14]. Besides macrophages, T-cells have been detected in atherosclerotic plaques albeit at low quantities [36]. Moreover, T cell function has been found to be strongly regulated by TGFß [37]. In a well-founded experimental approach, the groups of Goyova and co-workers [16] and Robertson et al. [17] have investigated whether the atheroprotective effects of TGFß1 may depend on intact TGFß signalling in T-cells. Interestingly, the most prominent effect of T cell specific abrogation of TGFß signalling in T cells of atherosclerotic mice as found by Robertson and co-workers [17] was a significant acceleration of atherosclerosis, whereas the group of Goyova [16] observed a decrease of lesion size in combination with a plaque phenotype more vulnerable to rupture. Although these two studies found opposite effects on lesion size, they were in general agreement that blockade of TGFß signalling in T cells increases vascular inflammation (which itself is certainly atherogenic). However, we must be aware that the immunological phenotype of transgenic mice with T-cell specific overexpression of a dominant negative type II TGFß receptor differs in dependence of the transcriptional control elements used and is strongly different to that of mice with a T cell specific TGFBR2 gene defect [18].

Given the validity of investigating T-cell specific TGFß1 signalling on atherogenesis, our present study was aimed to further clarify the role of TGFß1 and T-cells for this disease by using a murine overexpression model. To this end we used CD2-TGFß1 transgenic mice, overexpressing the TGFß1 cytokine in T-cells and exhibiting increased frequencies of CD4+ CD25+ regulatory T cells (Treg) in peripheral lymphoid organs and in the thymus [20], [21]. In this context it is important to mention that it is well established that Treg cells can decrease atherosclerosis (for review see [38]).

At a first glance, the fact that we failed to detect any influence of TGFß1 overexpression on disease progression in mice on WTD does not argue against a role of T cell specific TGFß1 on atherogenesis because the WTD 1) was previously reported to switch the T cell phenotype from the strongly atherogenic Th1-driven to a Th2-dominated response [39] and 2) highlights the role and function of innate immune cells (i.e. macrophages) which may overshadow a potential effect of the adaptive immunity in such an accelerated model of hypercholesterolemia [40]. To rule out this possibility, we also examined atherosclerotic lesion formation in CD2-TGFß1 ApoE^−/−^ and isogenic ApoE^−/−^ control mice on normal chow diet (ND). However, even under such a tempered diet regimen, we were not able to detect significant differences, neither at 16 nor at 24 weeks on ND. These results have to be interpreted against the background of previous essential studies on the role of lymphocytes and in particular Tregs in atherosclerotic lesion development. Investigating immunodeficient mice with targeted disruption in both ApoE and Rag1 (E0/R0), Dansky et al. showed that lymphocytes are not necessary for the formation of fibroproliferative plaques and play only a minor role in the rate of forming foam cell lesions [41]. More precisely, chow-fed E0/R0 mice had a 2-fold decrement in aortic root lesion size at 16 weeks of age. In contrast, there were no differences in either aortic root lesion size or the percent of the total aorta occupied by lesions in a second group of animals fed a WTD. In our own study, paucity of atherosclerotic lesion development and high inter-individual variations at 8 weeks on the chow-diet ruled out sufficient statistical analyses at this early time point.

In Rag1^−/−^ LDLR^−/−^ mice, Song et al. reported that after 8 weeks but not after 12 and 16 weeks on WTD, lesion development was significantly reduced in these mice compared with matched LDLR^−/−^ controls concluding that lymphocytes are important in early atherosclerosis [40]. Using different quantitative techniques, similar results have been reported in Rag2/ApoE-deficient mice: Daugherty et al. observed similar atherosclerotic lesion development compared with control ApoE-deficient mice after 12 weeks on the WTD [42]. By comparing the results of these three studies obtained at very early stages of lesion induction with each other and with our own study, one has to bear in mind the different animal models (ApoE-knockout versus LDLR-knockout) and the fact that Rag-deficient mice have a complete and sustained deficiency of both T and B lymphocytes. Increasing evidence concerning immunoglobulins in atherosclerotic lesions, circulating autoantibodies to modified low density lipoproteins in both humans and animals [43] as well as the adventitial immune response in atherosclerosis [44] suggests an important contribution of the latter to the pathogenesis of atherosclerosis.

In our study, a representative quantification of the total number of CD3-positive T-lymphocytes yielded no significant differences in atherosclerotic lesions of CD2-TGFß1 ApoE^−/−^ mice and isogenic ApoE^−/−^ controls after 24 weeks on WTD. The observed total number of T lymphocytes is in line with previous observations in ApoE-deficient mice [41], [42], [45] and suggests that T cell-specific overexpression of TGFß1 does not affect the T lymphocyte population in general. Taken together, our data suggest that T cells including Treg cells in the ApoE^−/−^ animal model do not contribute significantly to the role of TGFß1 on atherogenesis, which is obviously mediated by other cell types. We have recently shown that overexpression of TGFß1 in macrophages reduces and stabilizes atherosclerotic plaques in ApoE^−/−^ mice [14]. The synopsis of these data and our current results on TGFß1 overexpressing T cells suggests that potential effects of TGFß1 on atherosclerosis are most probably mediated by macrophages rather than T cells.
